# Serum 8-Oxo-dG as a Predictor of Sensitivity and Outcome of Radiotherapy and Chemotherapy of Upper Gastrointestinal Tumours

**DOI:** 10.1155/2018/4153574

**Published:** 2018-05-23

**Authors:** Ali Pour Khavari, Yongping Liu, Ellen He, Sven Skog, Siamak Haghdoost

**Affiliations:** ^1^Department of Molecular Biosciences, The Wenner-Gren Institute, Stockholm University, Svante Arrhenius vag 20C, 10691 Stockholm, Sweden; ^2^Clinical Oncology Laboratory, Department of Oncology, Changzhou Tumour Hospital Affiliated to Suzhou University, Honghe Road No. 68, Xinbei Area, Changzhou 213032, China; ^3^Sino-Swed Molecular Bio-Medicine Research Institute, Shenzhen, China; ^4^CIMAP-LARIA, University of Caen Normandy, Campus Jules Horowitz, Bd. Henri Becquerel, 14076 Caen, France

## Abstract

The level of oxidative stress is important in the initiation and progression of various age-related diseases, such as cancer. The level of oxidative stress may also play a significant role in cancer patients' response to treatment. We aimed to investigate whether serum 8-oxo-dG as a marker of oxidative stress is a predictor of tumour response. We used modified ELISA with a two-step filtration to analyse 8-oxo-dG in serum. The relationship between 8-oxo-dG levels, tumour response, and toxicity was studied in 19 oesophageal cancer patients who received radiotherapy and 16 gastric cancer patients who received chemotherapy. In the radiotherapy and the merged radio- and chemotherapy groups, the baseline levels of 8-oxo-dG were significantly lower in responder patients than in nonresponder patients and the increments after treatment were greater. In comparison with patients whose serum 8-oxo-dG levels decrease after treatment, patients with increasing levels had a longer median “progression-free survival.” Our results, although preliminary, suggest that serum levels of 8-oxo-dG may potentially be used to predict the sensitivity and outcome of radiotherapy and chemotherapy of upper gastrointestinal tumours. Patients with 8-oxo-dG levels that are low prior to treatment and subsequently increase after treatment may be more likely to benefit from the therapy.

## 1. Introduction

Oesophageal and gastric cancers are significant causes of morbidity and mortality. In 2012, gastric cancer was estimated to be the fifth most common cancer worldwide and the third leading cause of cancer death. While oesophageal cancer is less common than gastric cancer, its high mortality rate places it as the sixth leading cause of cancer-related deaths worldwide. Risk factors such as heavy smoking, genetic background, heavy alcohol consumption, certain food types, and hot beverages increase the risk of oesophageal and gastric cancer. In general, both oesophageal and gastric cancers are associated with poor prognosis despite improvements to their treatments by including advanced diagnostic and therapeutic methods [1, 2]. Prognostic biomarkers may play a significant role in choosing efficient treatments, reducing side effects, and improving quality of life. Currently, the main treatments for oesophageal and gastric cancers are surgery, radiotherapy, and chemotherapy, separately or in combination. The cytotoxic effects of radiotherapy and chemotherapy are mediated partly by the induction of reactive oxygen species (ROS), which leads to oxidative stress. Due to their unpaired electrons, ROS are unstable and react with other molecules, for example, lipids, proteins, RNA, DNA, dNTP, and NTP, modifying their structures and functions. Consequently, the functions of the cell become compromised. One of the frequently studied oxidative DNA base damages is 8-hydroxy-2′-deoxyguanosine (8-oxo-dG). 8-Oxo-dG is produced when ROS, for example, hydroxyl radicals, react with guanine in DNA or deoxyguanosine triphosphate (dGTP) in the cytoplasm. We have previously shown that the origin of extracellular 8-oxo-dG is the cytoplasmic content of dGTP which can be converted into 8-oxo-dGTP by free radical attack [3–5]. 8-Oxo-dG is excreted into extracellular environments such as urine, blood, and saliva. Elevated oxidative stress can partly explain the characteristics of cancer cells, for example, genomic instability, elevated proliferation rate, chemotherapy resistance, and metastasis [6].

Several publications show that levels of oxidative stress, measured with 8-oxo-dG in urine or blood serum, can be a useful biomarker for the response to radiation therapy and chemotherapy in cancer patients [7–9]. Additionally, several research groups have shown that the levels of 8-oxo-dG and MTH1 protein, an 8-oxo-dGTPase, play important prognostic roles in oesophageal as well as in gastric cancers [10–12]. However, the commercial ELISA kits available for detection of extracellular 8-oxo-dG are not specific [13], and other chromatographic-based methods, for example, high-pressure liquid chromatography (HPLC) with electrochemical detection or liquid chromatography with mass spectrometry detection (LC-MS/MS), are expensive, require sophisticated equipment and highly skilled personnel, and cannot be routinely used in the oncological clinics. Therefore, we have set up a sensitive and specific method where we first filter the serum sample on a Bond Elut column to isolate a fraction containing 8-oxo-dG and then use this fraction to determine 8-oxo-dG by a modified ELISA [3, 14].

We have previously reported that the levels of radiation-induced urinary and serum 8-oxo-dG could be used as a marker for determining individual radiation sensitivity in breast as well as in head and neck cancer patients [15–17]. In the present study, we investigated the relationship between serum levels of 8-oxo-dG and the therapy outcome of oesophageal and gastric cancers as well as therapy-related side effects. Blood samples from patients were collected at two time points: (1) before the start of oncological treatment and (2) two weeks after completed treatment. The levels of 8-oxo-dG in their blood serum were measured using our modified ELISA method.

## 2. Materials and Methods

### 2.1. Patients

Thirty-five patients with malignant tumours (stages III to IV) were recruited during March 2015 to July 2015 and underwent radiotherapy or chemotherapy at Changzhou Tumour Hospital. Patients with two different diagnoses were studied: 19 with oesophageal and 16 with gastric cancer. All patients had measurable lesions. The total group of patients is composed of 29 males and 6 females, ranging in age from 34 to 79 years, with a median age of 66 years. None of them had received previous radiotherapy or chemotherapy. The tumours were histologically and/or cytologically confirmed. The clinical stage of the tumours was confirmed based on the results of examination by X-ray, computed tomography (CT), magnetic resonance imaging (MRI), and other imaging examinations. All patients had an Eastern Cooperative Oncology Group (ECOG) performance status of ≤2, adequate bone marrow reserve, normal liver function, normal heart function, and normal kidney function. Furthermore, the patients had no history of prior malignancy. The study was performed in accordance with the ethical standards and approved by the Chinese Ethical Committee at Changzhou Tumour Hospital (Dnr 2015SY-001-01).

### 2.2. Treatments

Among the patients, 19 patients with local advanced oesophageal cancer received radiotherapy with 6 MeV photons. The planning target volume (PTV) encompassed the primary tumour site and a margin of approximately 1.5 cm. The prescription doses of PTV were 60 Gy in 30 fractions over a period of six weeks. 16 gastric cancer patients were treated with standard chemotherapy drug combination: folinic acid, fluorouracil, and oxaliplatin (FOLFOX4) 4 times during 2 weeks.

### 2.3. Response Evaluation

Tumour response was assessed according to the response evaluation criteria in solid tumours (RECIST) [18]: a complete response (CR) was defined as a complete disappearance of all objective evidence of disease, a partial response (PR) was defined as at least a 30% decrease in the sum of the longest diameters (LD) of tumour taking the baseline sum diameters as the reference, progressive disease (PD) was defined as a 20% increase in the sum of the LD of tumours or the appearance of one or more new lesions, and stable disease (SD) was defined as neither sufficient shrinkage to qualify for PR nor sufficient increase to qualify for PD. In this study, patients who achieved a CR or PR were classified as responders and all remaining patients were considered nonresponders.

### 2.4. Follow-Up

Physical examinations, measurement of carcinoembryonic antigen CA199 and thymidine kinase 1 (marker of proliferation) levels, and whole-body computed tomography were carried out every 3 months in the first year and every 6 months thereafter to evaluate therapy response. Progression-free survival (PFS) was the time from study entry until disease progression, death, or the day of the last follow-up visit, whichever came first. The Radiation Therapy Oncology Group (RTOG) scoring schema [19] was used to evaluate the acute radiation-related toxicity to the chest wall skin and oesophageal mucosa. In order to facilitate statistical analysis, we classified patients displaying side effects of RTOG grades 2, 3, and 4 as the moderate or severe group. The toxicity of chemotherapy was graded according to NCI-CTC, version 4.0 (available at http://www.oncology.tv/SymptomManagement).

### 2.5. ELISA Measurement of Serum 8-Oxo-dG

3 ml of blood samples was collected in tubes without anticoagulant. After about two hours, the blood serum was isolated by centrifuging the tubes at 250 ×g for 20 minutes. 8-Oxo-dG was measured using a modified competitive ELISA as described previously [3, 4, 16]. All samples were coded and analysed blindly. The ELISA kit was provided by Health Biomarkers Sweden AB. Briefly, 800 *μ*l blood serum was filtered using a C18 solid phase extraction Bond Elut column (Varian, CA) as described previously [14]. The filtration step is necessary to remove products other than 8-oxo-dG that could cross-react with the monoclonal antibody. The purified samples were lyophilised and reconstituted to 1 ml by adding PBS. The filtration step was repeated once more. 90 *μ*l of the purified samples was processed further for the detection of 8-oxo-dG according to the protocol provided by the company. The absorbance of the samples was read at 450 nm using an automatic ELISA plate reader. Each sample was analysed in triplicate, and the samples belonging to one individual patient were analysed using the same 96-well ELISA plate. A standard curve for 8-oxo-dG (0.05–10 ng/ml) was established for each plate covering the range of 8-oxo-dG in the samples. Validation of the modified ELISA method was performed by HPLC-EC during the previous study [4]. Comparisons between the modified ELISA and the HPLC-EC methods showed a linear correlation at the concentration range found in human blood serum (*r*^2^ = 0.87, *p* < 0.05). There was no correlation between ELISA and HPLC-EC results when unfiltered samples were used.

### 2.6. Measurement of Serum TK1

The concentration of serum TK1 (STK1p) was measured using a commercial kit based on an improved chemiluminescent (ECL) dot blot assay as described by the manufacturer (SSTK Ltd., Shenzhen, China). 3 *μ*l samples of serum was directly applied to a nitrocellulose membrane in duplicate. The serum samples were probed with an anti-TK1 chicken IgY antibody raised against a peptide (residue 195-225, GQPAG PDNKE NCPVP GKPGE AVAAR KLFAPQ). The TK1 peptide was dotted at different concentrations (2.2, 6.6, and 20 pM) as a quality control standard. The intensities of the spots on the membrane were determined by the CIS-l Imaging System (SSTK Ltd., Shenzhen, China). From the intensities of the TK1 quality control standards of known concentrations, the concentration of STK1p was calculated and expressed as pM. For a detailed description of the STK1p assay, see Chen et al. [20]. Within this study, 2.0 pM of STK1p was used as a risk threshold value.

### 2.7. Measurement of Serum CA199

Serum CA199 levels were measured using the ADVIA Centaur® XP automated Immunoassay System (Siemens Healthcare Diagnostics, Erlangen, Germany) according to the manufacturer's protocol. Levels of serum CA199 ≥ 37 U/ml were considered abnormal.

### 2.8. Statistical Methods

For the statistical analysis, the paired sample *t*-test and the independent sample *t*-test were used. The chi-square test was used to determine correlations between the changes of 8-oxo-dG concentration and the responses of tumour to treatments or acute side effects. Kaplan-Meier survival curves and the log-rank test were used to analyse univariate distributions for progression-free survival. A *p* value lower than 0.05 was considered to indicate a significant difference.

## 3. Results

### 3.1. Associations between Serum 8-Oxo-dG and Clinicopathological Features

In the merged group of patients, the baseline 8-oxo-dG concentrations were significantly associated with the degree of tumour differentiation. Patients with highly differentiated tumours had higher 8-oxo-dG in the serum (0.48 ± 0.30 ng/ml) in comparison to patients with poorly or undifferentiated tumours (0.25 ± 0.17 ng/ml) (Table 1). Serum 8-oxo-dG was significantly related to the tumour marker CA199 and to gender, but not to TK1 (Table 1).

### 3.2. Associations between Changes of Serum 8-Oxo-dG Level and Tumour Response

To investigate if changes of serum 8-oxo-dG level are associated with tumour response, we divided the patients into 2 groups: those who showed objective response (CR + PR) and individuals with no response (PD + SD), respectively. Additionally, the association between tumour response and 8-oxo-dG was investigated in the patients treated with radiotherapy (oesophageal cancer) or chemotherapy (gastric cancer) as separate groups and as one merged group. The changes of 8-oxo-dG concentrations are summarised in Table 2. We found that the changes of serum 8-oxo-dG were associated with the tumour response. In Table 3, the levels of 8-oxo-dG in the serum are presented for each therapy group based on their response before and 2 weeks after treatment. As shown in Table 3, the levels of 8-oxo-dG increased in the merged group, CR + PR, from 0.21 to 0.38 ng/ml (*p* < 0.05). The increases for radiotherapy patients were from 0.25 to 0.43 ng/ml (*p* < 0.05) and for chemotherapy patients from 0.17 to 0.27 ng/ml (nonsignificant). For SD + PD groups, a decrease or no change of 8-oxo-dG was found when the levels of 8-oxo-dG after treatment were compared to the baseline levels. To explore whether baseline serum 8-oxo-dG level could predict the outcome of radiotherapy and chemotherapy, we analysed the relationship between baseline serum 8-oxo-dG and tumour response in different treatment groups. The overall results presented in Table 3 indicate that the CR + PR groups have lower baseline 8-oxo-dG in comparison to the SD + PD groups. These observations suggest that patients with low baseline levels of serum 8-oxo-dG that then increase markedly after treatment are more sensitive to treatment.

### 3.3. Associations between Changes of Serum 8-Oxo-dG Level and Side Effects of Radiotherapy or Chemotherapy

To investigate if changes of serum 8-oxo-dG level are associated with acute side effects, we compared the serum 8-oxo-dG concentrations before and after treatment in patients with severe side effects to the concentrations in the patients that displayed almost no side effects. In the radiotherapy group, we found that an increase in serum 8-oxo-dG level was closely related to acute skin reactions and acute oesophageal mucosa reactions (*p* = 0.047 and 0.018, resp.). In the chemotherapy group, patients with an increase in serum 8-oxo-dG appeared to be more prone to suffer from 3 or 4 degrees of bone marrow suppression and 3 or 4 degrees of gastrointestinal reactions when compared to patients with declining concentrations, although the difference between the groups did not reach statistical significance (*p* = 0.09 and *p* = 0.21, resp.) (Table 4).

### 3.4. Associations between Serum 8-Oxo-dG Levels and Progression-Free Survival

**Figure 1:**
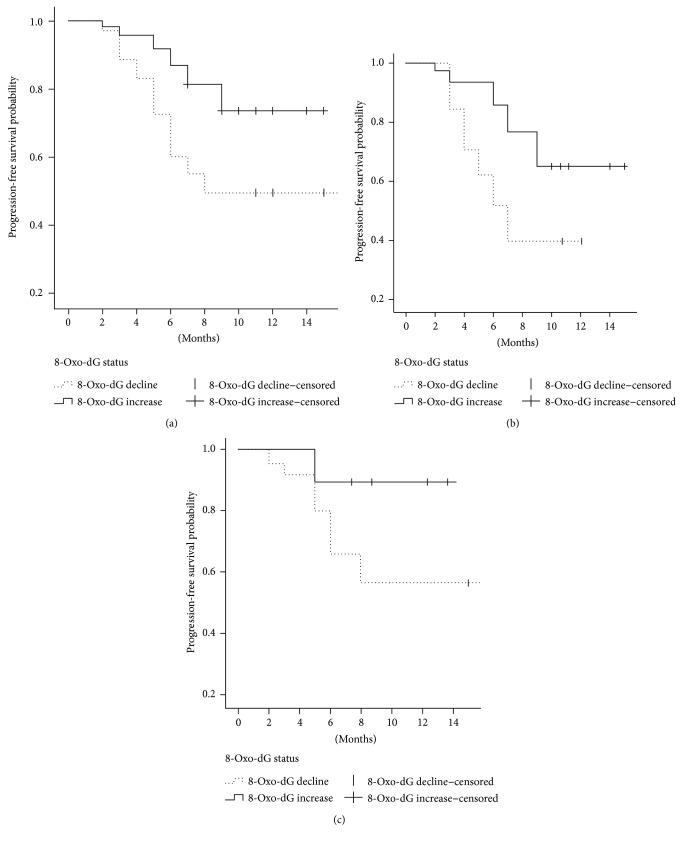
Kaplan-Meier survival curves for all patients (a), radiotherapy treatment (b) and chemotherapy treatment (c) with serum 8-oxo-dG level decline or increase. Censored values indicate patients who died and patients without disease progression.

The median PFS was 9 months (range, 2–17 months).  Figure 1(a) shows the Kaplan-Meier survival curves for all patients with serum 8-oxo-dG level decline or increase. The changes in the serum concentration of 8-oxo-dG were calculated by subtracting the baseline concentration before treatment from the concentration of serum 8-oxo-dG two weeks after the radiotherapy or chemotherapy. Compared to the patients whose serum 8-oxo-dG declined, the patients with increasing serum 8-oxo-dG concentration had a significantly longer median PFS (median PFS, no defined versus 8 months). Figures 1(b) and 1(c) show the Kaplan-Meier survival curves for patients who received radiotherapy and chemotherapy, respectively. When compared to patients whose serum 8-oxo-dG declined, chemotherapy-treated patients with increasing serum 8-oxo-dG concentrations had significantly longer median PFS, while the difference did not reach statistical significance for the radiotherapy group (median PFS, no defined versus 7 months in the radiotherapy group and no defined versus no defined in the chemotherapy group, *p* < 0.086 and 0.001, resp.).

The median time of follow-up was 9 months. Some of the patients underwent follow-up at 17 months. During the study period, 4 of the 35 patients (11.4%) deceased. As a result of the low death rate, a meaningful determination of association between serum 8-oxo-dG level and death was not possible to perform.

## 4. Discussion

Within the present investigation, we used a modified ELISA method for the detection of 8-oxo-dG in blood serum of the patients. Extracellular 8-oxo-dG originates from 8-oxo-dGTP in the nucleotide pool when free radicals react with dGTP. MTH1 hydrolyses 8-oxo-dGTP to 8-oxo-dGMP. 8-Oxo-dGMP is further dephosphorylated, and 8-oxo-dG is released from the intra- to the extracellular milieu where it can be detected and used as a marker for oxidative stress [3, 4]. We found that the baseline levels of 8-oxo-dG in the serum of responder patients (chemo- and radiotherapy—CR + PR) were significantly lower compared to the levels in nonresponder patients (SD + PD) (0.21 ± 0.15 ng/ml and 0.52 ± 0.34 ng/ml, resp.). When breaking down the results into radiotherapy and chemotherapy groups, we still observe a significantly lower level of 8-oxo-dG in the CR + PR group as compared to the SD + PD group for radiotherapy patients (0.25 ± 0.14 and 0.46 ± 0.25 ng/ml, *p* = 0.048), while for the chemotherapy group, the level of 8-oxo-dG was only nonsignificantly lower in the CR + PR than in the SD + PD group (0.17 ± 0.16 and 0.37 ± 0.32 ng/ml, resp.). The data indicates that patients with lower 8-oxo-dG in the serum before the start of oncological treatment have better prognosis than patients with higher 8-oxo-dG levels suggesting that baseline levels of 8-oxo-dG in the serum of oesophageal and gastric cancer patients may have a prognostic value.

These results are also in accord with previously published data on ovarian, renal, and hepatocellular carcinoma as well as lung cancer [8, 14, 21, 22]. It was shown that there is an association between 8-oxo-dG levels, tumour size, and clinical stage in renal cell carcinoma.

**Figure 2:**
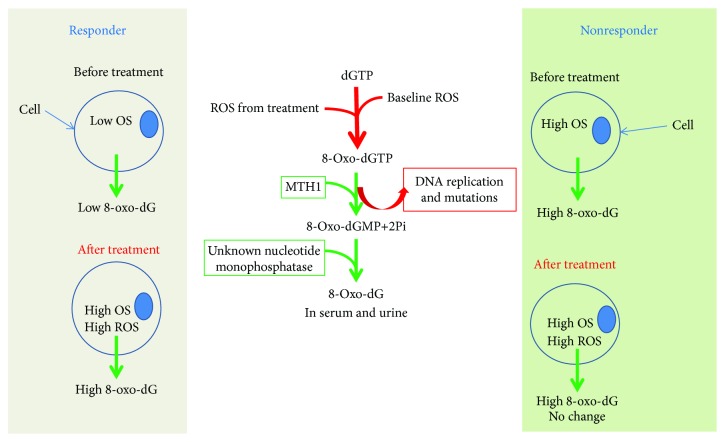
Schematic picture of the obtained results and possible mechanisms comparing a cell from a responder with that from a nonresponder. OS: oxidative stress.

Another interesting finding is the observation of the changes in the levels of 8-oxo-dG after the treatments (Table 3). The results show that the levels of 8-oxo-dG increase significantly in the CR + PR groups, almost 2-fold, after the treatment compared to the baseline levels. On the contrary, the levels of 8-oxo-dG in the SD + PD groups (all patients and radiotherapy and chemotherapy groups) did not increase after the treatments and may have decreased. It should be noted that the baseline level of 8-oxo-dG is higher in the SD + PD group, indicating that the SD + PD patients are already at a state of stress and their cells cannot cope with additional ROS induced by the treatments. The high baseline levels of oxidative stress in the SD + PD groups may have various causes including higher metabolic activity due to large tumour burden, tumour aggressiveness, and type of tumours. In this case, exposed cells may die and not produce additional 8-oxo-dG. In contrast, patients in the CR + PR groups have lower oxidative stress; therefore, they can cope with and excrete elevated 8-oxo-dGTP induced by the treatments. A summary of the results and some suggested mechanisms that have been introduced in the discussion are presented in Figure 2.

Individual 8-oxo-dG levels before and after treatment could have a predictive value when considering the side effects of the treatments. The data presented in Table 4 indicates that radiotherapy-treated patients, whose 8-oxo-dG levels are increased after therapy, are at increased risk of severe skin reactions and severe mucosa reactions, while in comparison with the chemotherapy groups, no correlations between 8-oxo-dG levels and acute side effects were observed.

The results presented here indicate that it might be possible to distinguish the CR + PR group from the SD + PD group considering the baseline 8-oxo-dG as well as changes of 8-oxo-dG levels after the treatment. However, there is an overlap between these groups indicating that 8-oxo-dG analysis alone cannot predict sensitivity to treatment. We plan to study other factors, for example, expression of oxidative stress proteins to investigate whether combinations of protein expression with excretion of 8-oxo-dG would better predict treatment response.

CA199 is a tumour-related biomarker mainly used for monitoring the efficiency of tumour treatment, but not for diagnosis of malignant diseases. TK1 is a proliferation biomarker useful for the prognosis of recurrence and survival of malignant patients and also for early discovery of premalignancy and malignancy in health screening [23, 24]. While CA199 and 8-oxo-dG correlated with one another, TK1 did not correlate with 8-oxo-dG, indicating that 8-oxo-dG in serum may not correlate with tumour proliferation rate, but instead with the presence of tumour in the body. In this respect, CA199 behaves like 8-oxo-dG and did not correlate with serum TK1. This is in agreement with previous studies of tumour patients [23]. Thus, it should be remembered that different tumour-related biomarkers reflect different properties of a tumour and should be used in combination.

The aim of cancer treatment is to optimise the probability of tumour control while minimising the unwanted acute or late side effects. However, since there is no test for individual prediction of radio- and chemosensitivity available for clinical use, the maximal dose delivered is adapted to those few sensitive patients who show severe side effects. Therefore, developing predictive tests would allow for the majority of the cancer patients to be treated with higher doses, hopefully leading to improved tumour control and reduced side effects.

Our previous research indicates that extracellular 8-oxo-dG could be used as a biomarker to predict the sensitivity of cancer patients to radiotherapy when considering the side effects [15–17]. We established the dose-response relationship for radiation-induced oxidative stress using 8-oxo-dG in blood serum after irradiation of whole blood samples. The blood samples were collected from breast cancer as well as head and neck cancer patients, all with varying degrees of posttreatment side effects. The patients were grouped into radiosensitive and normo-sensitive groups based on their acute or late radiotherapy-induced side effects, for example, acute skin reaction in breast cancer and osteoradionecrosis in head and neck cancer patients. We found that the dose response for radiation-induced 8-oxo-dG in serum differs between radiosensitive and normo-sensitive patients. In the normo-sensitive groups, the baseline levels of 8-oxo-dG were lower in comparison to those in the radiosensitive groups, and furthermore, the increments after treatment were larger. However, 2-3 weeks after start of radiotherapy the level of 8-oxo-dG in urine of the sensitive patients increased and reached a level similar to that in normo-patients. This data may indicate that the radiosensitive patiients excrete 8-oxo-dG in a slower manner. Using a proteomic approach, we could show that several antioxidant proteins, for example, SOD1, PRDX2, and PARK7, were downregulated in the normo-sensitive patients and some antioxidant proteins, for example, BLVRB and PRDX2, were upregulated in the radiosensitive breast cancer patients [16, 25]. These results indicated that the expression of antioxidant proteins as well as oxidative stress levels play important roles in the sensitivity of breast cancer and “head and neck” cancer patients to radiotherapy. For the prediction of the effect of chemotherapy, further investigations are required. Consistent with previous studies [9], we found a significant prognostic value for 8-oxo-dG in serum for oesophageal and gastric cancers. Patients whose baseline 8-oxo-dG levels are low and increase after treatment may be more likely to benefit from radiotherapy or chemotherapy. Our findings are new but should be considered preliminary due to the low number of patients involved within this study.

## Figures and Tables

**Table 1:** Associations between serum 8-oxo-dG level and characteristics of the study cohorts.

Type	Mean (ng/ml)	±SD	*n*	*p* value
Age (median age: 66 years)				
≥66	0.28	0.19	21	
<66	0.33	0.29	14	0.511
Gender				
Male	0.26	0.18	29	
Female	0.48	0.36	6	0.049
CA199				
≥37 U/ml	0.44	0.35	9	
<37 U/ml	0.24	0.15	26	0.027
TK1				
≥2 pmol/ml	0.30	0.20	22	
<2 pmol/ml	0.29	0.25	13	0.954
Differentiation				
Highly or moderately differentiated	0.48	0.30	8	
Poorly differentiated or undifferentiated	0.25	0.17	27	0.011

**Table 2:** Associations between changes of serum 8-oxo-dG and tumour response.

Changes of serum 8-oxo-dG	Tumour response		*χ* ^2^	*p* value
	CR + PR	SD + PD		
All patients (*n* = 35)				
Increase	11	3		
Decline	6	15	8.41	0.006
The radiotherapy group (*n* = 19)				
Increase	7	1		
Decline	4	7	4.96	0.026
The chemotherapy group (*n* = 16)				
Increase	4	2		
Decline	2	8	3.48	0.062

**Table 3:** Concentrations of serum 8-oxo-dG before and after the treatment.

Types	All patients (ng/ml)		Radiotherapy group (ng/ml)		Chemotherapy group (ng/ml)	
	CR + PR	SD + PD	CR + PR	SD + PD	CR + PR	SD + PD
Before treatment	0.21 ± 0.15	0.52 ± 0.34^☆^	0.25 ± 0.14	0.46 ± 0.25^☆☆^	0.17 ± 0.16	0.37 ± 0.32
After treatment	0.38 ± 0.32^∗^	0.35 ± 0.23	0.43 ± 0.33^∗∗^	0.33 ± 0.17	0.27 ± 0.24	0.39 ± 0.36

^∗^ *p* = 0.04 and ^∗∗^*p* = 0.05 compared with the values before treatment; ^☆^*p* = 0.013 and ^☆☆^*p* = 0.048 compared with the values in the CR + PR groups.

**Table 4:** Associations between changes of serum 8-oxo-dG and acute side effects.

Acute side effects	Changes of serum 8-oxo-dG		*χ* ^2^	*p* value
	Increase	Decline		
Radiotherapy group (*n* = 19)				
Moderate or severe skin reaction				
Yes	3	0		
No	6	10	3.96	0.047
Moderate or severe oesophageal mucosa reaction				
Yes	4	0		
No	5	10	5.63	0.018
Chemotherapy group (*n* = 16)				
3 or 4 degrees of bone marrow suppression				
Yes	3	2		
No	2	9	2.79	0.09
3 or 4 degrees of gastrointestinal reaction				
Yes	3	3		
No	2	8	1.57	0.21

## Data Availability

The data used to support the findings of this study are available from the corresponding author upon request.
